# Clinical activity of pembrolizumab with or without chemotherapy in advanced pulmonary large-cell and large-cell neuroendocrine carcinomas: a multicenter retrospective cohort study

**DOI:** 10.1186/s12885-023-10952-w

**Published:** 2023-05-16

**Authors:** Lianxi Song, Fei Zhou, Tian Xu, Liang Zeng, Qing Xia, Zhan Wang, Li Deng, Yizhi Li, Haoyue Qin, Huan Yan, Zhe Huang, Jinye Mi, Qinqin Xu, Nong Yang, Caicun Zhou, Yongchang Zhang

**Affiliations:** 1 Department of Medical Oncology, Lung Cancer and Gastrointestinal Unit, Hunan Cancer Hospital/The Affiliated Cancer Hospital of Xiangya School of Medicine, Central South University, Changsha, 410013 China; 2 Graduate Collaborative Training Base of Hunan Cancer Hospital, Hengyang Medical School, University of South China, Hengyang, 421001 China; 3 Department of Medical Oncology, Yiyang Center Hospital, Yiyang, 413000 China; 4 Department of Medical Oncology, Shanghai Pulmonary Hospital, Tongji University School of Medicine, Shanghai, 200433 China; 5 Department of Oncology, The Second Xiangya Hospital, Central South University, Changsha, 410013 China; 6 Department of Oncology, State Key Laboratory for Oncogenes and Related Genes, Shanghai Cancer Institute, Renji Hospital, Shanghai Jiao Tong University School of Medicine, Shanghai, 200002 China; 7 Department of Medical Oncology, Qinghai Provincial People’s Hospital, Xining, 810007 China

**Keywords:** Pulmonary large cell/neuroendocrine carcinoma, Immune checkpoint inhibitor, PFS

## Abstract

**Background:**

Immune checkpoint inhibitors (ICI)-based combination strategies have improved the survival outcomes in advanced non-small cell lung cancers; however, data regarding their efficacy remains limited for uncommon histological types, including large-cell carcinoma (LCC) and large-cell neuroendocrine carcinoma (LCNEC).

**Methods:**

We retrospectively analyzed a total of 60 patients with advanced LCC and LCNEC – 37 treatment-naïve and 23 pre-treated – who received pembrolizumab with or without chemotherapy. Treatment and survival outcomes were analyzed.

**Results:**

Of the 37 treatment-naïve patients who received first-line pembrolizumab combined with chemotherapy, the 27 patients with LCC had an overall response rate (ORR) of 44.4% (12/27) and a disease control rate (DCR) of 88.9% (24/27); whereas 10 patients with LCNEC had an ORR of 70% (7/10) and DCR of 90% (9/10). The median progression-free survival (mPFS) was 7.0 months (95% confidence intervals [CI]: 2.2–11.8) and median overall survival (mOS) was 24.0 months (95%CI: 0.0–50.1) for first-line pembrolizumab plus chemotherapy of LCC (*n* = 27), whereas mPFS was 5.5 months (95%CI: 2.3–8.7) and mOS was 13.0 months (95%CI: 11.0–15.0) for first-line pembrolizumab plus chemotherapy of LCNEC (*n* = 10). Of the 23 pre-treated patients who received subsequent-line pembrolizumab with or without chemotherapy, mPFS was 2.0 months (95% CI: 0.6–3.4) and mOS was 4.5 months (95% CI: 0.0–9.0) for LCC and mPFS was 3.8 months (95% CI: 0.0–7.6) and mOS was not reached for LCNEC.

**Conclusion:**

Our study provides real-world clinical evidence of the anti-tumor activity of pembrolizumab plus chemotherapy in advanced LCC and LCNEC, indicating that this regimen could serve as a treatment option, particularly as first-line therapy, for improving the survival outcomes of patients with these rare histological subtypes of lung cancer.

**Trial registration:**

NCT05023837(ESPORTA, 27/08/2021).

**Supplementary Information:**

The online version contains supplementary material available at 10.1186/s12885-023-10952-w.

## Highlights

• Rare lung cancer subtypes, LCC and LCNEC, benefits from immunochemotherapy.

• Disease control of LCC and LCNEC is achieved with 1L pembrolizumab plus chemotherapy.

• 1L pembrolizumab plus chemotherapy imparts better survival outcomes for LCC/LCNEC.

**Supplementary Information:**

The online version contains supplementary material available at 10.1186/s12885-023-10952-w.

## Introduction

Large-cell carcinoma (LCC) and large-cell neuroendocrine carcinoma (LCNEC) are among the rare histological subtypes of lung cancers [1]. These types of tumors tend to be highly aggressive, associated with early metastasis, and resistance to platinum-based chemotherapy regimens [2–4]. As a result, patients often have poor prognoses, with an overall survival between 8–16 months [4, 5]. Immune checkpoint inhibitors (ICI) in combination with chemotherapy have demonstrated superior efficacy in treating common lung cancer histology types, including lung adenocarcinoma harboring no actionable mutations, squamous cell lung carcinoma, and small-cell lung cancer [6–10]. However, clinical evidence on the efficacy of ICI combined with chemotherapy in treating rare histological types of lung cancers is limited to case reports and case series [11–13]. Since LCC and LCNEC are often excluded from prospective clinical trials, there is a lack of clinical trial data to assess the anti-tumor activity of such combination regimens in these rare lung cancer histological subtypes. A previous study reported the high expression of programmed death ligand 1 (PD-L1) in some rare cancers [14]. These findings suggested the potential sensitivity of patients with rare cancer types to ICI-based combination therapies, which, however, has not yet been adequately evaluated [15]. Herein, we report the results of a retrospective analysis conducted on patients with either LCC or LCNEC who received ICI-based regimens that are pooled from three institutes. We assessed the efficacy of pembrolizumab combination therapy or monotherapy in the first-line or later-line treatment setting.

## Methods

### Patient inclusion criteria

We retrospectively screened 380 patients who were histologically diagnosed with either LCC or LCNEC between January 1, 2018 and September 30, 2021, and received treatment at any of the three hospitals namely: Hunan Cancer Hospital, Second Xiangya Hospital, or Shanghai Pulmonary Hospital. The following were the study inclusion criteria: (1) Histologically diagnosed stage IIIB-IV LCC or LCNEC; (2) Not detected with inhibitor-sensitizing mutations in genes, including *EGFR*, *ALK*, *ROS1*, *RET*, *ERBB2*, *MET*, and *BRAF*. Patients detected with *KRAS* mutation were included; and (3) Received at least two cycles of pembrolizumab with or without chemotherapy and had data for at least one tumor assessment. This study was retrospectively registered as a clinical trial in clinicaltrials.gov (NCT05023837; ESPORTA, 27/08/2021). The protocol of this study was approved by the Hunan Cancer Hospital Institutional Ethics Committee. Waiver of consent was approved by the institutional review board. All procedures in our study were performed in accordance with the ethical standards of the institutional and national research committees and the 2013 revision of the Declaration of Helsinki. The data cutoff date was February 10, 2022.

### Histological classification of LCC and LCNEC and PD-L1 assessment

Histological subtype was independently reviewed and classified by two pulmonary pathologists in each institution according to the 4th edition of the World Health Organization (WHO) Classification of Lung Tumors (2015) [1]. Based on the WHO classification, surgical specimens are needed for more accurate identification of LCC and LCNEC features [1]. Thus, the histopathological diagnosis for some specimens was established using surgically resected specimens, whenever available. Nevertheless, considering clinical practice, large-cell morphological features may also be identified on needle biopsy specimens as the presence of bulky and polygonal cells, poorly differentiated, pulmonary origin and excluding the possibility of any other pathological subtypes through immunohistochemical characterization with neuroendocrine antibodies such as chromogranin A (CgA) and synaptophysin (Syn) to distinguish them from other subtypes of non-small cell carcinoma [1, 5]. The diagnostic criteria for LCNEC include (1) cytomorphologic features of non-small cell carcinoma (ie, large cells, abundant cytoplasm, prominent nucleoli), (2) neuroendocrine architecture (ie, organoid nesting, trabecular growth pattern, peripheral palisading, and/or rosette-like structures) with focal to diffuse immunohistochemical staining for one or more neuroendocrine markers, and (3) high proliferation rate as shown by > 10 mitoses per 2 mm^2^ with necrosis that is often geographic [16–19]. Using the diagnostic criteria enumerated above, the diagnosis of LCNEC in biopsy specimens becomes more feasible and practicable owing to the recent requirement for obtaining larger volumes of tissue during the biopsy [17, 19, 20]. Figures S1-S2 show representative images of hematoxylin–eosin staining and immunohistochemistry for LCC and LCNEC. The distribution of the methods for obtaining tissue specimens is presented in Table 1.

**Table 1:** Clinical characteristics of the 60 patients with pulmonary large-cell carcinoma (LCC) or pulmonary large-cell neuroendocrine carcinoma (LCNEC) who received pembrolizumab with or without chemotherapy

Characteristic	Total, n (%)	Subgroup	
		LCC	LCNEC
No. of patients	60	42	18
Age, years			
Median	61.5	61.3	61.9
Range	31–79	31–79	47–76
Sex			
Male	56(93.3)	39(92.9)	17(94.4)
Female	4(6.7)	3(7.1)	1(5.6)
Smoking history			
Never smoker	13(21.7)	9(21.4)	14(77.8)
Former smoker	47(78.3)	33(78.6)	4(22.2)
Biopsy method			
Surgery	18(30.0)	16(38.1)	2(11.1)
Lung biopsy	30(50.0)	18(42.9)	12(66.7)
Bronchoscopy biopsy	12(20.0)	8(19.0)	4(22.2)
ECOG performance status			
0–1	58(96.7)	41(97.6)	17(94.4)
≥ 2	2 (3.3)	1(2.4)	1(5.6)
Stage			
IIIb/IIIc	9(15.0)	6(12.3)	3(16.7)
IV	51(85.0)	36(85.7)	15(83.3)
Brain metastasis			
Yes	12(20.0)	10(23.8)	2(11.1)
No	48(80.0)	32(76.2)	16(88.9)
PD-L1 expression (*N* = 44)			
< 1%	19(43.2)	13(38.2)	6(60.0)
1%-49%	13(29.5)	9(26.5)	4(40.0)
≥ 50%	12(27.3)	12(35.3)	0
Treatment line			
First-line	37(61.7)	27(64.3)	10(55.6)
≥ Second-line	23(38.3)	15(35.7)	8(44.4)
Treatment regimens			
Pembrolizumab monotherapy	6(10.0)	4(9.5)	2(11.1)
Chemo + Pembrolizumab	54(90.0)	38(90.5)	16(88.9)

*Abbreviation*: *ECOG* Eastern Cooperative Oncology Group

PD-L1 immunohistochemistry was assessed using clone 22C3. The percentage of positive tumor cells was counted by experienced and qualified pathologists and presented as tumor proportion score (TPS).

### Treatment regimen and assessment of treatment outcomes

Pembrolizumab was administered intravenously with a fixed dose of 200 mg. Tumor response was assessed according to the Response Evaluation Criteria in Solid Tumors (RECIST) version 1.1. Treatment responses were assessed at baseline (Day 0) and after every 2 cycles (approximately 45–50 days) using computed tomography or magnetic resonance imaging. The radiological images were reviewed by two radiologists. Objective response rate (ORR) refers to the proportion of patients whose disease responded to the treatment as shown by at least a 30% decrease in the size of the target lesions. Disease control rate (DCR) refers to the proportion of patients whose disease was evaluated as complete response, partial response and stable disease. Progression-free survival (PFS) was calculated from the time pembrolizumab (alone or with chemotherapy) was administered until confirmation of progressive disease (PD), death, or last follow-up. Overall survival (OS) was calculated from the time pembrolizumab (alone or with chemotherapy) was administered until death or the last follow-up.

### Statistical analysis

Categorical variables were summarized as frequencies and percentages and compared using the chi-square or Fisher's exact test as appropriate. Kaplan–Meier method was applied for survival estimation. 95% confidence intervals (CIs) for PFS and OS were calculated by Cox survival model. All statistical analyses were performed using SPSS software (version 24).

## Results

Of the 380 patients diagnosed with LCC and LCNEC, 60 patients received pembrolizumab with or without chemotherapy and were included in our analysis (Fig. S3). Patient characteristics were summarized in Table 1. The patients had a median age of 61.5 (range 31–79) years, with 93.3% (56/60) males and 78.3% (47/60) were former or current smokers. Our cohort comprised 70.0% patients with LCC (*n* = 42) and 30.0% with LCNEC (*n* = 18). All 37 patients who received the regimen in the first-line setting were administered pembrolizumab plus pemetrexed and carboplatin, while the remaining 23 patients (38.3%) were treated with pembrolizumab monotherapy (*n* = 6) or combined with chemotherapy (*n* = 17) as second or later-line therapy. PD-L1 (22C3) status was assessable in 44 patients, which comprised 19 patients identified with < 1% PD-L1 expression, 13 patients had 1–49% PD-L1 expression, and 12 patients had > 50% PD-L1 expression (Table 1).

**Fig. 1:**
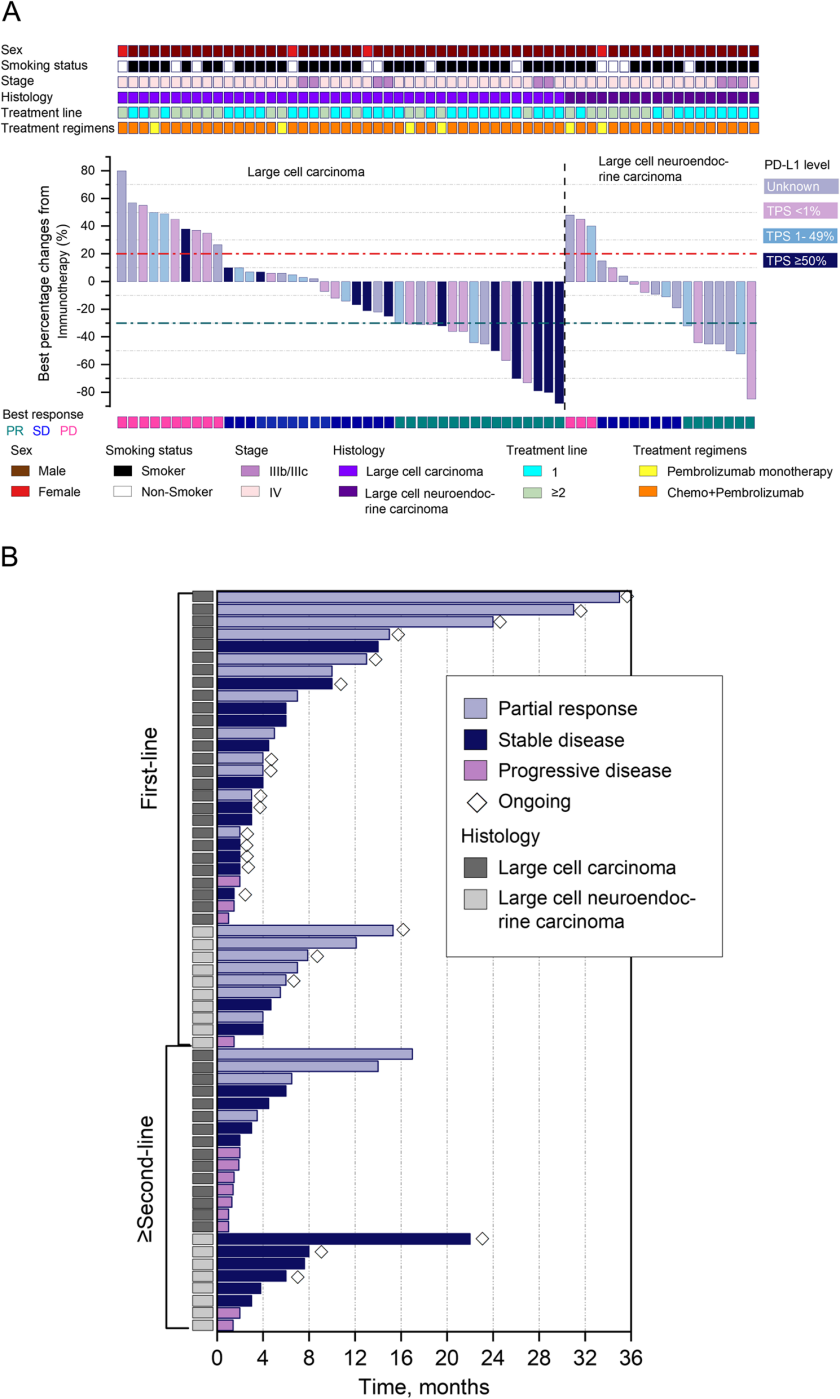
Treatment outcomes with pembrolizumab with or without chemotherapy for pulmonary large-cell carcinoma (LCC; *n* = 42) and large-cell neuroendocrine carcinoma (LCNEC; *n* = 18). **A** Waterfall plot of the maximum percent change in tumor size of target lesions from baseline. The patients were grouped according to histological subtypes. Clinical details of each patient were annotated and represented by various colors. Red dashed line denotes the threshold of 20% increase in tumor size relative to baseline and evaluated as progressive disease (PD). Blue dashed line denotes the threshold of 30% reduction in tumor size relative to baseline and evaluated as partial response (PR); **B** Swimmer plot showing the time on treatment, which is calculated starting on the day treatment is initiated until disease is evaluated as progressive disease (PD). The patients were grouped according to treatment setting. The histological subtype and best response were indicated by colors. Diamond shapes indicate the patients who are still receiving treatment at data cut-off. The median follow-up of the cohort is 12 months

**Fig. 2:**
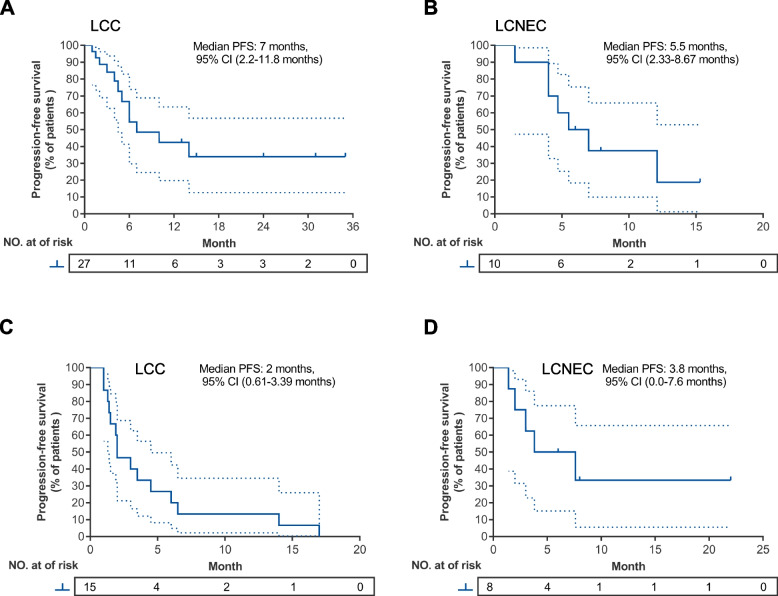
Progression-free survival (PFS) for pembrolizumab with or without chemotherapy. Kaplan–Meier curves illustrating the PFS of patients with large-cell carcinoma (LCC) (**A**, **C**) or large-cell neuroendocrine carcinoma (LCNEC) (**B**, **D**) who received pembrolizumab with chemotherapy as first-line therapy (**A-B**) or pembrolizumab with or without chemotherapy as second-line or later-line therapy (**C-D**). The dashed lines correspond to the 95% lower and upper confidence intervals. Tick marks indicate the number of censored cases per time point. The risk table below shows the number of cases per time point

**Fig. 3:**
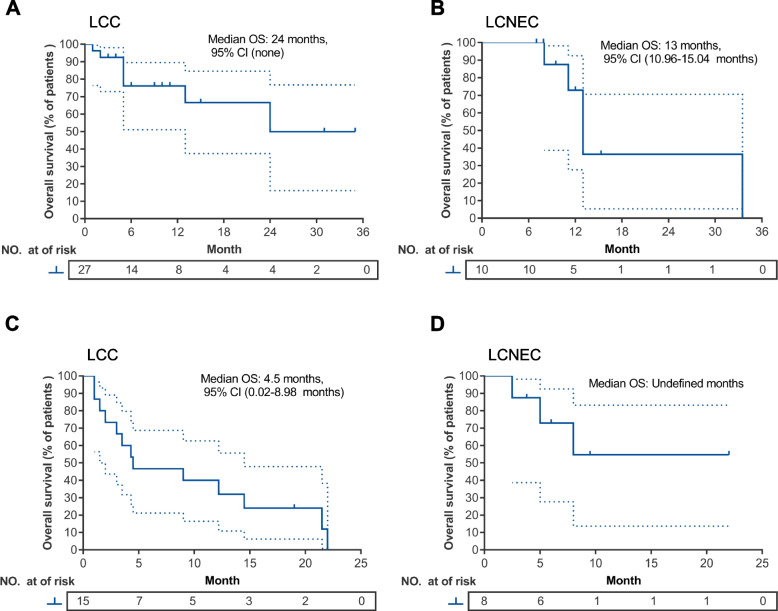
Overall survival (OS) for pembrolizumab with or without chemotherapy. Kaplan–Meier curves illustrating the OS of patients with large-cell carcinoma (LCC) (**A**, **C**) or large-cell neuroendocrine carcinoma (LCNEC) (**B**, **D**) who received pembrolizumab with chemotherapy as first-line therapy (**A**, **B**) or pembrolizumab with or without chemotherapy as second-line or later-line therapy (**C**, **D**). The OS was computed from the initiation of pembrolizumab-containing regimen until death or last follow-up. Dashed lines correspond to the 95% lower and upper confidence intervals. Tick marks indicate the number of censored cases per time point. The risk table below shows the number of cases per time point

Table S1 summarizes the detailed characteristics, tumor mutational status, PD-L1 expression (if available), treatment information, and clinical outcomes, including PFS and OS of each patient. Table S2 summarizes the detailed treatment history of the 23 patients who received pembrolizumab with or without chemotherapy as subsequent-line therapy. According to subtype, objective response rate (ORR) and disease control rate (DCR) were 44.4% (12/27) and 88.9% (24/27) for patients with LCC and 70% (7/10) and 90% (9/10) for patients with LCNEC who received pembrolizumab with chemotherapy as first-line therapy (Fig. 1A-B); whereas ORR and DCR were 26.7% (4/15) and 53.3% (8/15) for patients with LCC and 0% (0/8) and 75% (6/8) for patients with LCNEC who received pembrolizumab with or without chemotherapy as second-line or subsequent-line therapy (Fig. 1A-B). Compared with patients who received pembrolizumab-containing regimen as first-line therapy regardless of histology, DCR was significantly lower in patients who received the regimen as second-line or later-line therapy (86.5% vs. 60.9%, *p* = 0.02; Fig. S4). Among the patients treated with first-line pembrolizumab with chemotherapy, the median PFS was 7.0 months (95% CI: 2.2–11.8) for patients with LCC (*n* = 27; Fig. 2A) and 5.5 months (95% CI: 2.3–8.7) for patients with LCNEC (*n* = 10; Fig. 2B). Second-line or later-line treatment with pembrolizumab with or without chemotherapy yielded a median PFS of 2.0 months (95% CI: 0.6–3.4) for patients with LCC (*n* = 15; Fig. 2C) and 3.8 months (95% CI: 0.0–7.6) for patients with LCNEC (*n* = 8; Fig. 2D). The median OS among the patients who received first-line pembrolizumab with chemotherapy was 24.0 months (95% CI: not reached) for those with LCC (Fig. 3A) and 13.0 months (95% CI: 11.0–15.0) for those with LCNEC (Fig. 3B). The median OS among the patients who received later-line pembrolizumab with or without chemotherapy was 4.5 months (95% CI: 0.0–9.0) for those with LCC (Fig. 3C) and not reached (95% CI: not reached) for those with LCNEC (Fig. 3D).

**Fig. 4:**
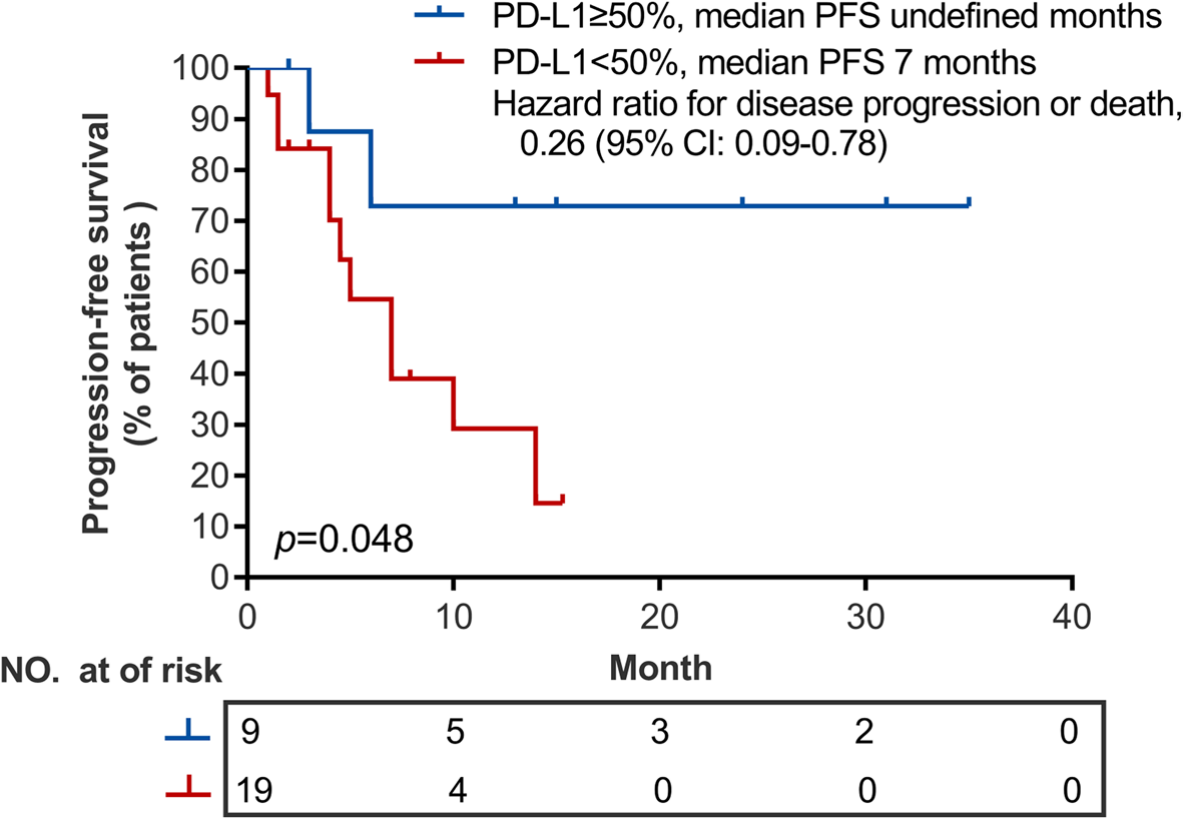
Kaplan–Meier curve comparing the progression-free survival of 28 patients who received pembrolizumab with chemotherapy as first-line therapy stratified according to PD-L1 expression status as high (≥ 50%; *n* = 9) and low (< 50%; *n* = 19)

We further analyzed the association between PD-L1 expression status and PFS in patients who received pembrolizumab with chemotherapy as first-line therapy (*n* = 28) regardless of subtype. Interestingly, PFS was significantly longer for patients with PD-L1 expression ≥ 50% (*n* = 9) than those who had PD-L1 expression < 50% (*n* = 19) (not reached vs 7.0 months; Hazard ratio: 0.26, 95% CI: 0.09–0.78; *p* = 0.048, Fig. 4). PFS was comparable for patients with LCC (*n* = 27) and LCNEC (*n* = 10) who received first-line pembrolizumab plus chemotherapy (7.0 vs 6.25; *p* = 0.53; Fig. S5). Univariate analyses revealed that age younger than 60 years was associated with worse PFS (*p* = 0.004); while having PD-L1 expression levels ≥ 50% (*p* = 0.024) was associated with better PFS with first-line pembrolizumab plus chemotherapy. However, multivariate analyses did not reveal any variables associated with PFS (Fig. S6). These subgroup analyses were not performed for those who received pembrolizumab-containing regimens as second-line or later due to limited sample size. Figures S7 and S8 show the imaging results for representative cases of LCC (No.60) and LCNEC (No.59) who clinically benefitted and had remarkable tumor regression with first-line pembrolizumab plus chemotherapy.

## Discussion

The prognosis of LCC and LCNEC remains poor owing to the lack of optimal treatment strategies for managing these rare lung cancer subtypes [21]. The rarity of LCC and LCNEC poses as a major barrier to the conduct of prospective clinical trials and thus data on the efficacy of therapeutic options, particularly immunotherapy, in the first-line or later-line settings from a large cohort remains elusive. This retrospective study investigated the clinical activity of pembrolizumab with or without chemotherapy in the treatment of rare lung cancer histological subtypes, LCC and LCNEC. To our knowledge, our study reports the effectiveness of pembrolizumab with or without chemotherapy, regardless of treatment setting, in the largest cohort of patients with either LCC or LCNEC. Our study cohort had a predominance of males and smokers, which were consistent with the reported clinical features of patients with these rare lung cancer subtypes [22, 23]. High PD-L1 expression (TPS ≥ 50%) was detected in 27.3% of our cohort, which was in line with the frequency of PD-L1 overexpressing tumors (17.7%) reported by a previous study that evaluated the efficacy of ICI monotherapy in uncommon non-small cell lung cancer (NSCLC) subtypes [23].

Advanced LCC or LCNEC are associated with poor prognoses, with an observed PFS of 4.4–5.8 months with platinum doublet regimen and OS of only 8–12.6 months [4]. The combined use of pembrolizumab and chemotherapy as first-line therapy demonstrated better prognoses than chemotherapy only. Our data showed a median PFS of 7.0 months and a median OS of 24.0 months for patients who received first-line pembrolizumab plus chemotherapy. This data indicates that the addition of pembrolizumab in the first-line chemotherapy could improve not only the DCR but also PFS and OS outcomes of patients with LCC or LCNEC.

A study conducted by Komiya et al. reported improved OS outcomes with immunotherapy use (regardless of treatment setting) compared with non-use in patients with stage IV LCNEC (*n* = 37) (12-month survival rate, 34.0% vs 24.1%; 18-month survival rate, 29.1% vs 15.0%) [24]. A subgroup analysis of the CA209-538 clinical trial for rare cancers had shown promising clinical activity of ipilimumab and nivolumab combination in patients with advanced neuroendocrine tumors, including lung [25]. Similarly, the phase 2 trial GCO-001 NIPINEC has also reported encouraging outcomes with nivolumab and ipilimumab use in pre-treated patients with advanced, refractory LCNEC (*n* = 92) [26]. Promising treatment outcomes were also observed from smaller cohorts [27, 28]. Chauhan et al. reported three cases of LCNEC that received nivolumab treatment after progression from platinum-based chemotherapy and achieved durable response with a complete radiological response or stable disease [27]. Levra et al. reported an ORR of 60% and mPFS of 14.0 months with single-agent nivolumab or pembrolizumab after progression to platinum-based first-line chemotherapy from a small case series involving 10 pre-treated patients with advanced LCNEC [28]. The later-line usage of pembrolizumab among pre-treated patients was also analyzed in our study. Dudnik et al. reported the median OS in LCNEC calculated starting from either disease diagnosis or ICI initiation were 12.4 and 11.0 months, respectively (*n* = 41) as compared with an OS of 6.0 months among non-ICI-treated group (*n* = 84) [29]. In our cohort, we observed that second-line or later-line administration of pembrolizumab with or without chemotherapy could achieve a median OS of 9.0 months calculated from the time of initiating the pembrolizumab-containing regimen. The difference in response and prognosis may be related to having more patients with PD-L1-positive tumors (94.7%) and stage II or III disease (43.2%) included in the study by Dudnik et al., whereas our study cohort was comprised of patients with locally advanced/advanced disease.

Moreover, findings from our cohort have demonstrated promising treatment outcomes with ICI treatment despite low PD-L1 positivity. We speculate that the inherent genetic heterogeneity and higher tumor mutation burden (TMB) in rare histological subtypes such as LCNEC and LCC, which are associated with smoking history/status, may positively impact the efficacy of ICI-containing-regimens in this patient subset. TMB may indicate genomic instability and has been found to be positively associated with tobacco exposure among patients with NSCLC [30, 31]. Albeit lack of consensus on the TMB cutoff, increasing TMB levels were shown to be associated with immune cell infiltration and inflammatory T-cell-mediated response, which may result in increased sensitivity to ICIs in NSCLC regardless of PD-L1 expression [32]. Thus, the combination of TMB and smoking status has been proposed as a potential predictor of the efficacy of combination immunotherapy in advanced NSCLC [33]. Consistently, a case study has demonstrated a significant and durable response to pembrolizumab of a patient with relapsed stage IB LCNEC with PD-L1-negative tumor that was positive for PD-L1 amplification and high TMB [13]. Furthermore, molecular characterization of LCNEC and LCC has shown distinct mutational profiles with enrichment of *RB1/TP53* mutations in LCNEC, while genomic alterations in *SMARCA2, STK11, KEAP1,* and *MYCL1* were commonly detected in specimens from patients with LCC and LCNEC [34–40]. *KEAP1* mutations were reported as potential biomarkers for immunotherapy outcomes [41].

Immunotherapy, particularly ICI-containing combination strategies, has completely revolutionized cancer therapy by greatly improving the prognoses of patients regardless of cancer type. The interpretation of our findings is limited by the retrospective nature of our study. Some patients who submitted for NGS testing used a small 8-gene panel covering only the classic NSCLC oncogenic genes: *EGFR*, *ALK*, *BRAF*, *ERBB2*, *KRAS*, *MET*, *ROS1*, and *RET*, and did not interrogate *TP53* and *RB1*, which are genes commonly altered in LCNEC [36, 38–40].

Our study has provided real-world clinical evidence that pembrolizumab plus chemotherapy could be an attractive first-line treatment strategy in improving the survival outcomes of patients with advanced LCC or LCNEC, which could also be applicable to patients with other uncommon histology.

## Supplementary Information

**Additional file 1:** **Figure S1.** H&E stain of LCC and LCNEC. **Figure S2.** IHC stain of LCC and LCNEC. **Figure S3.** Study flow chart of patient selection. **Figure S4.** Disease control rate with first-line pembrolizumab. **Figure S5. **PFS of first-line pembrolizumab in LCC and LCNEC. **Figure S6.** Univariate and multivariate analysis for PFS in first-line pembrolizumab. **Figure S7.** Case vignette illustrating the treatment outcome of patient NO.60. **Figure S8.** Case vignette illustrating the treatment outcome of patient NO.59. **Table S1.** Detailed information of the 60 patients with large cell lung cancer or large cell neuroendocrine carcinoma who received pembrolizumab with or without chemotherapy. **Table S2.** Detailed treatment history for 23 patients with large cell carcinoma (LCC) or large cell neuroendocrine carcinoma (LCNEC) who received ≥second-line pembrolizumab with or without chemotherapy.

## Data Availability

The data sets used and analyzed for the current study are included as Supplementary Tables S1, S2. Other relevant data are available from the corresponding author on request.
