# Supplementary material for: Clinical activity of pembrolizumab with or without chemotherapy in advanced pulmonary large-cell and large-cell neuroendocrine carcinomas: a multicenter retrospective cohort study
Source: BMC Cancer. 2023 May 16;23:443. doi: 10.1186/s12885-023-10952-w (PMC10186661; doi:10.1186/s12885-023-10952-w)
Supplement: Supplementary file 1 — Additional file 1: Figure S1. H&E stain of LCC and LCNEC. Figure S2. IHC stain of LCC and LCNEC. Figure S3. Study flow chart of patient selection. Figure S4. Disease control rate with first-line pembrolizumab. Figure S5. PFS of first-line pembrolizumab in LCC and LCNEC. Figure S6. Univariate and multivariate analysis for PFS in first-line pembrolizumab. Figure S7. Case vignette illustrating the treatment outcome of patient NO.60. Figure S8. Case vignette illustrating the treatment outcome of patient NO.59. Table S1. Detailed information of the 60 patients with large cell lung cancer or large cell neuroendocrine carcinoma who received pembrolizumab with or without chemotherapy. Table S2. Detailed treatment history for 23 patients with large cell carcinoma (LCC) or large cell neuroendocrine carcinoma (LCNEC) who received ≥second-line pembrolizumab with or without chemotherapy. [file 12885_2023_10952_MOESM1_ESM.pdf]

Figure S1. H&E stain of LCC and LCNEC.

Representative pathology images of large-cell carcinoma (LCC) and large-cell neuroendocrine carcinoma (LCNEC). Hematoxylin-eosin staining of LCC at 20× (top left) and 40× (top right) magnification and of LCNEC at 20× (bottom left) and 40× (bottom right) magnification.

LCC

LCNEC

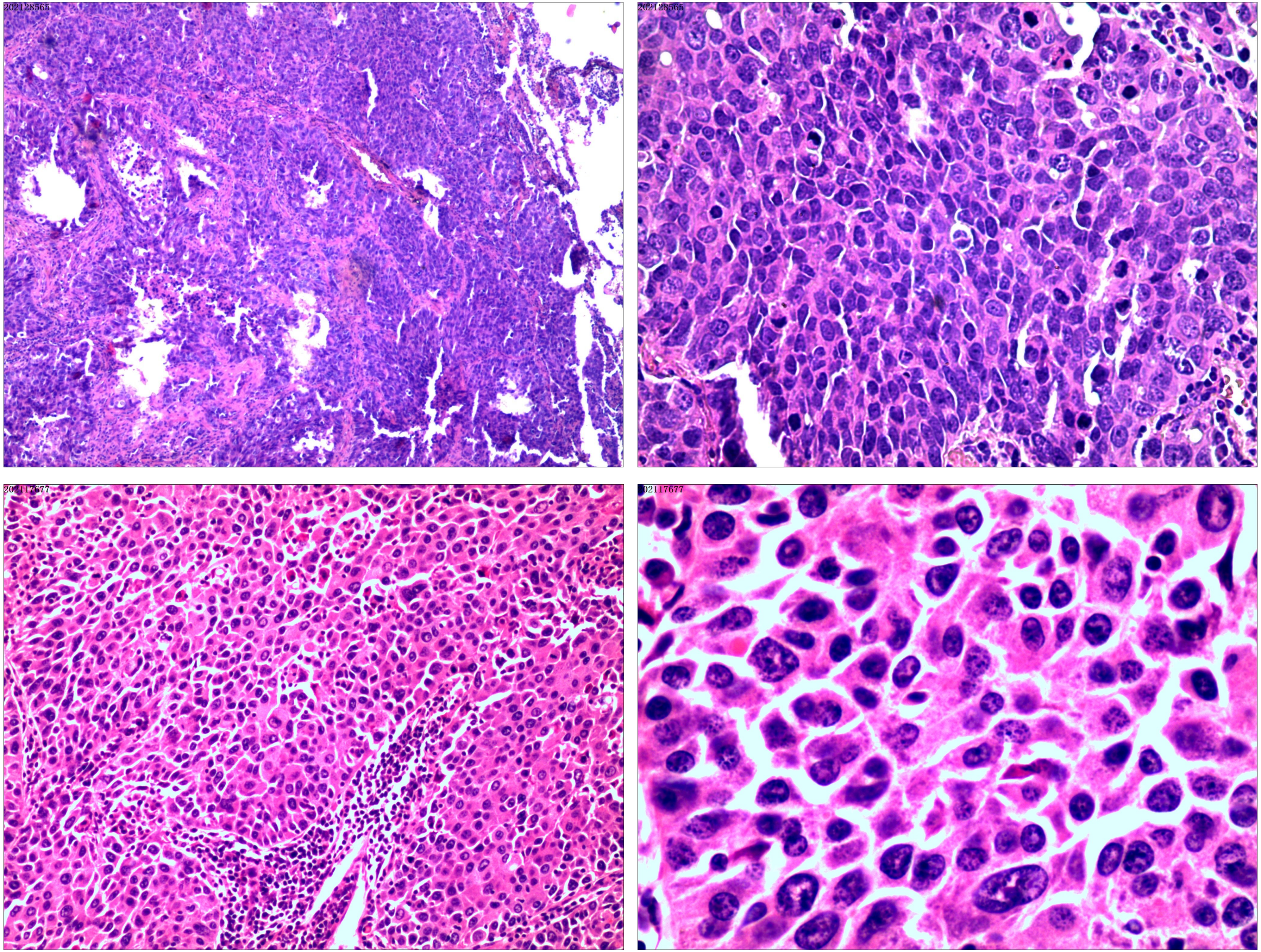

Figure S2. IHC stain of LCC and LCNEC.

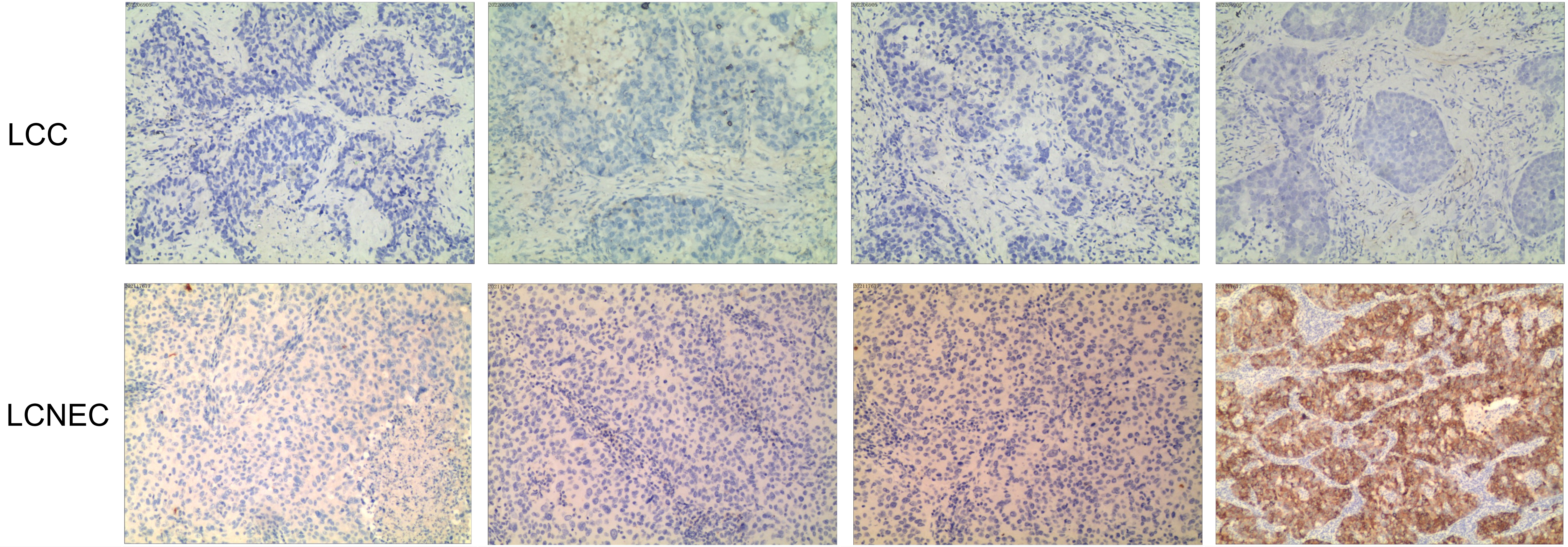

	P40	TTF-1	CgA	Syn
LCC	-	-	-	-
LCNEC	-	-	-	+++

Representative immunohistochemical (IHC) staining images for differential diagnosis of large-cell carcinoma (LCC; upper panels) and large-cell neuroendocrine carcinoma (LCNEC; lower panels). IHC was performed using LCC and LCNEC biomarkers, including P40, TTF-1, CgA, and Syn.

Figure S3. Study flow chart of patient selection.

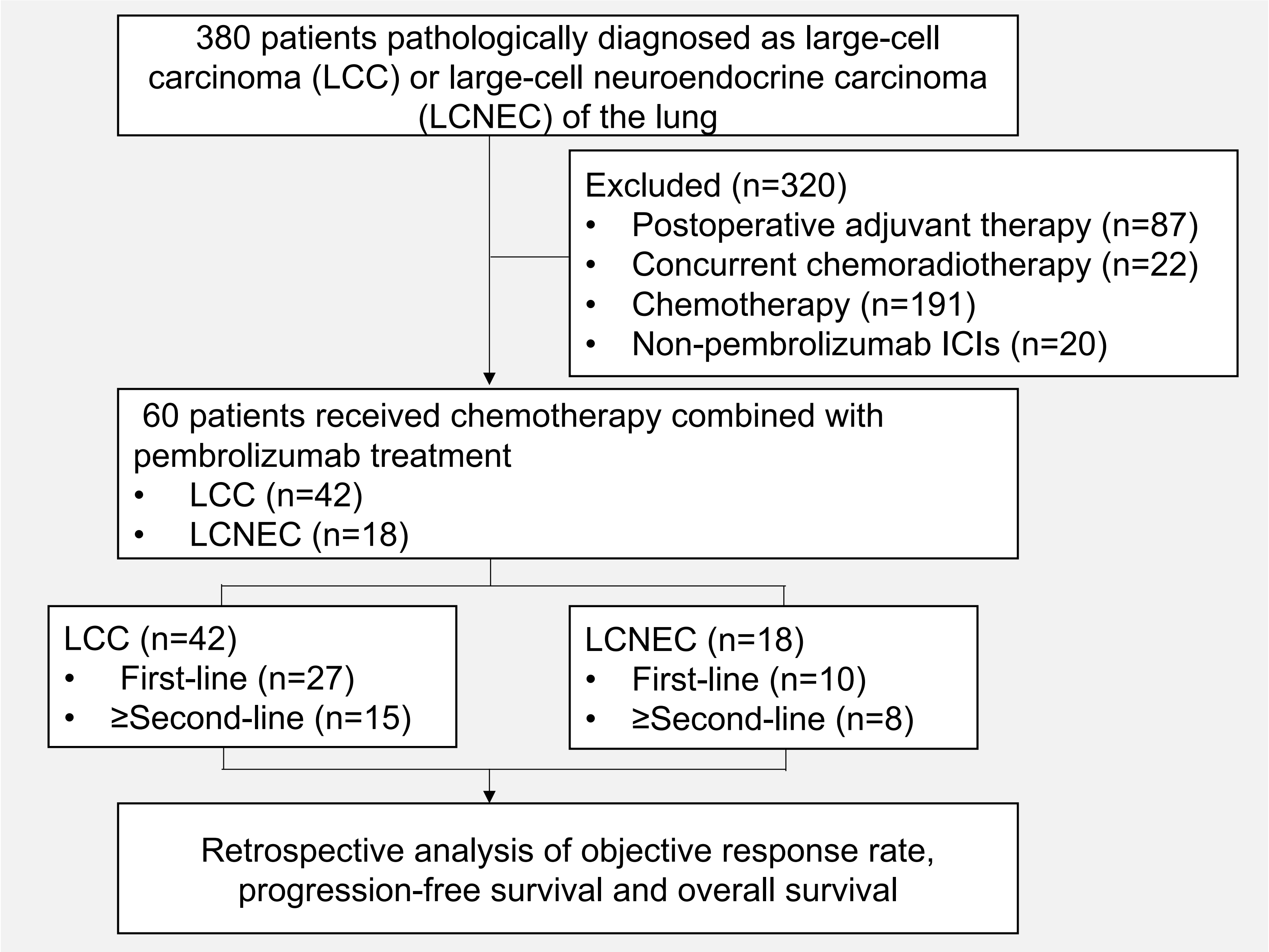

Figure S4. Disease control rate with first-line pembrolizumab.

Disease control rate for pembrolizumab combined with chemotherapy as first-line treatment (left bar) or pembrolizumab with or without chemotherapy as second or later-line treatment (right bar). PD, progressive disease; PR, partial response; SD, stable disease

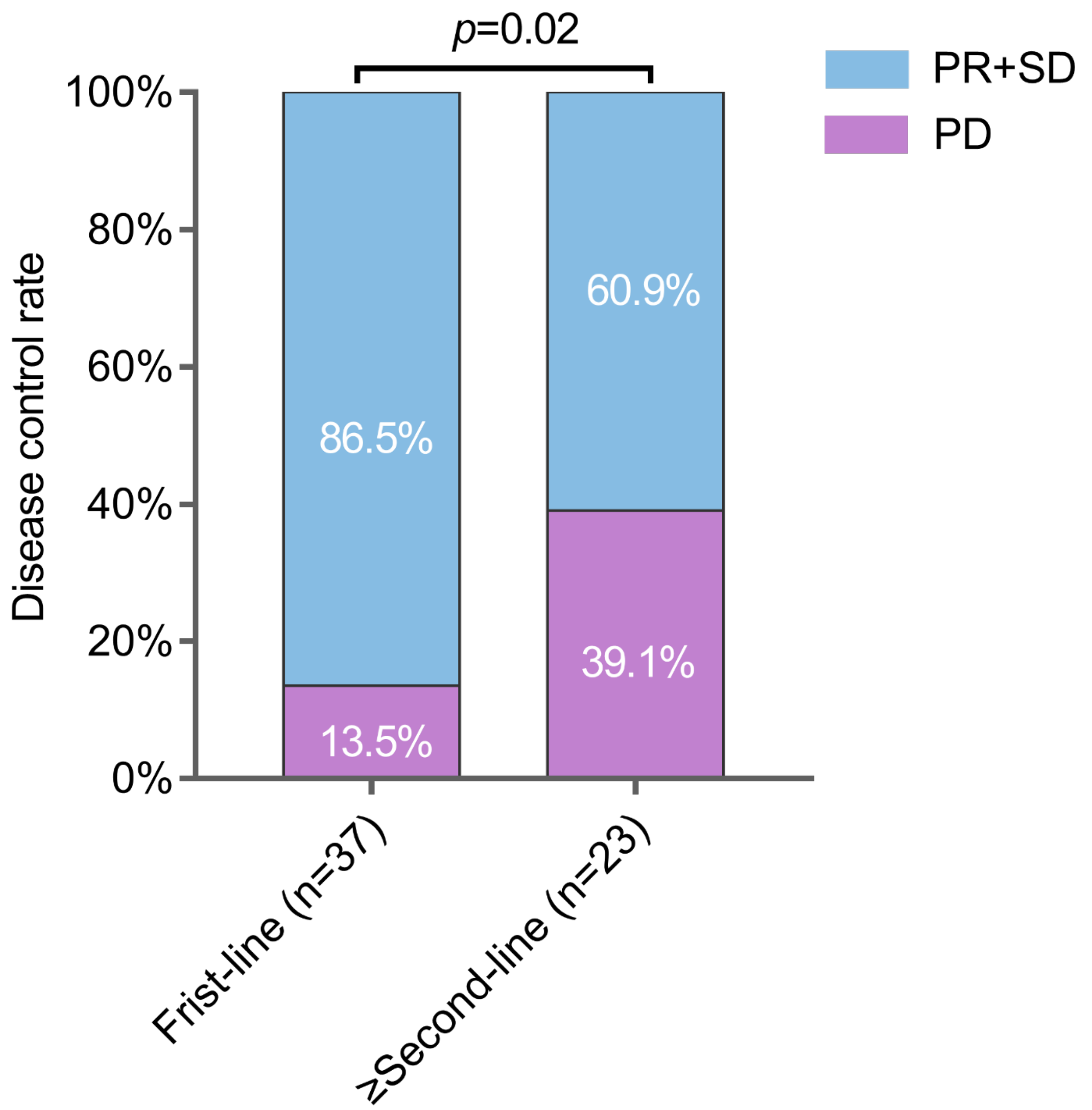

Figure S5. PFS of first-line pembrolizumab in LCC and LCNEC.

Kaplan-Meier curve comparing the progression-free survival (PFS) of patients who received pembrolizumab combined with chemotherapy as first-line treatment stratified according to histological subtypes (i.e., large-cell carcinoma [n=27] and large-cell neuroendocrine carcinoma [n=10]).

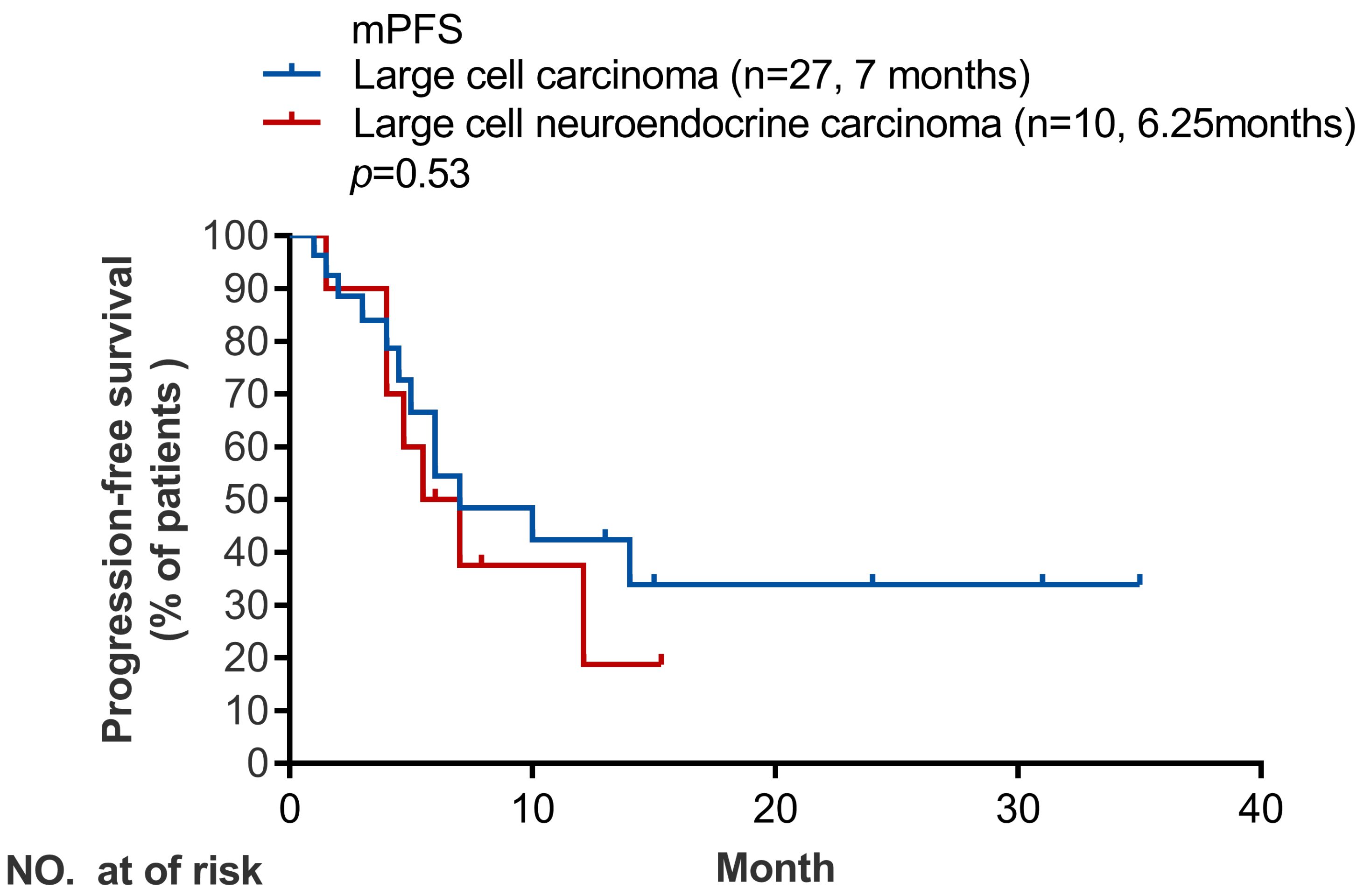

Large cell carcinoma	27	8	3	2	0
Large cell neuroendocrine carcinoma	10	2	0	0	0

Figure S6. Univariate and multivariate analysis for PFS in first-line pembrolizumab.

The univariate (left) and multivariate (right) analysis to analyze the impact of various clinical variables on the progression-free survival (PFS) of patients who received pembrolizumab combined with chemotherapy as first-line treatment (n=37).

Characteristics	Total(N)	HR(95% CI)	P value	HR(95% CI)	P value
Gender	37		0.791		0.318
Male	35	Reference		Reference	
Female	2	0.76 (0.10-5.77)	0.791	0.23 (0.01-4.13)	0.318
Age	37		0.004		0.149
>60	28	Reference			
≤ 60	9	4.20 (1.59-11.08)	0.004	3.60 (0.63-20.50)	0.149
Smoking history	37		0.923		0.607
Smoker	30	Reference			
Never smoker	7	1.05 (0.38-2.94)	0.923	1.78 (0.20-16.26)	0.607
Brain metastasis	37		0.474		0.388
Without	30	Reference			
With	7	0.63 (0.18-2.20)	0.474	0.37 (0.04-3.52)	0.388
Stage	37		0.187		0.442
IIIb/IIIc	9	Reference			
IV	28	2.30 (0.67-7.94)	0.187	2.07 (0.32-13.20)	0.442
Pathology	37		0.527		0.843
LCC	27	Reference			
LCNEC	10	1.35 (0.53-3.47)	0.527	0.85 (0.18-4.07)	0.843
Actionable alteration	37		0.402		0.728
Not identified	19	Reference			
TP53	6	2.52 (0.76-8.34)	0.131	0.62 (0.05-7.06)	0.701
KRAS	5	0.68 (0.15-3.14)	0.623	1.18 (0.17-10.26)	0.88
Unknown	7	1.11 (0.30-4.03)	0.878	1.59 (0.23-11.16)	0.642
PD-L1 level	37		0.109		0.223
<1%	9	Reference			
1-49%	10	0.52 (0.15-1.77)	0.293	0.980 (0.15-6.47)	0.982
≥50%	9	0.16 (0.03-0.78)	0.024	0.15 (0.012-1.91)	0.145
Unknown	9	0.98 (0.32-2.97)	0.971	1.15 (0.12-10.85)	0.904

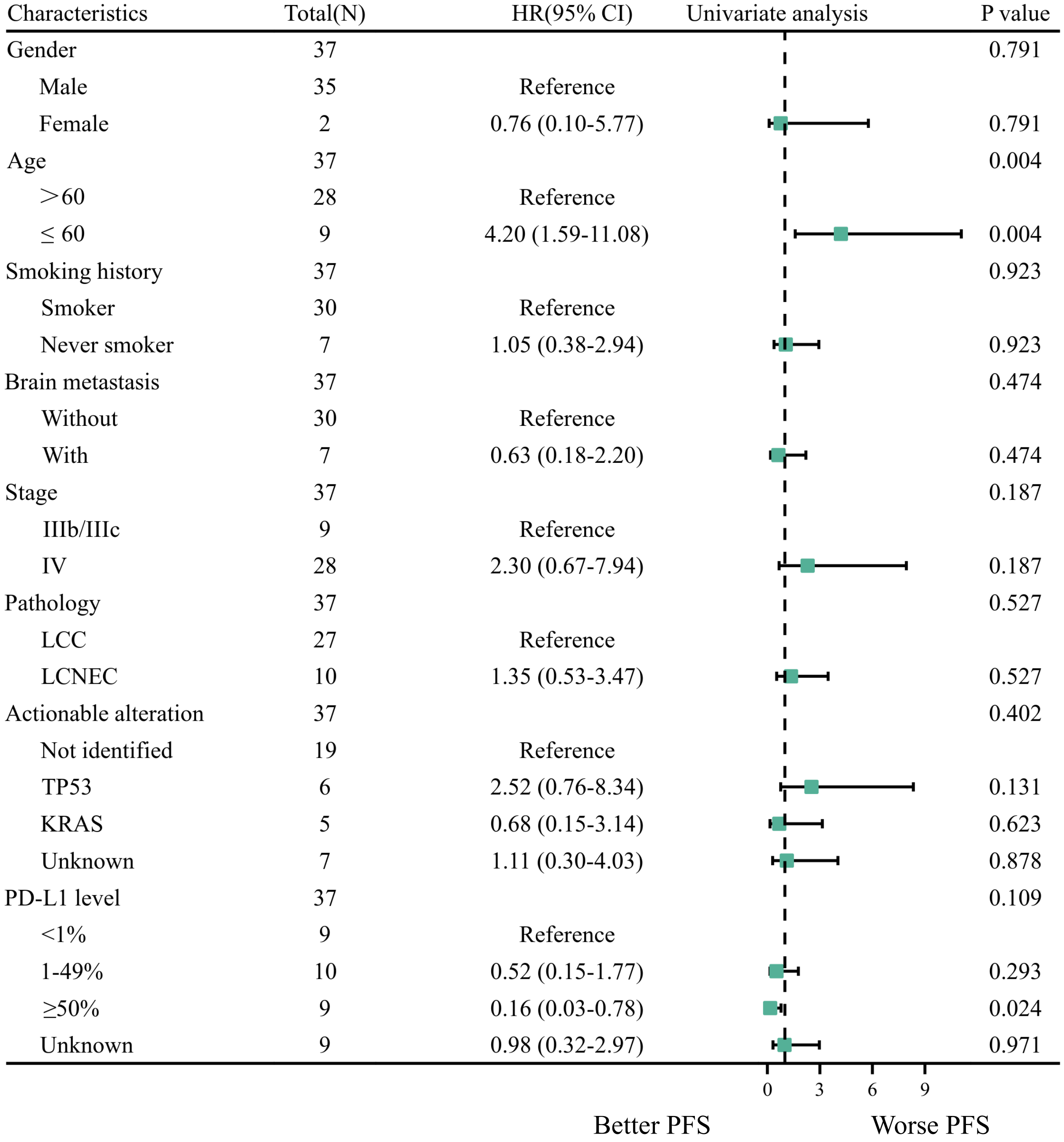

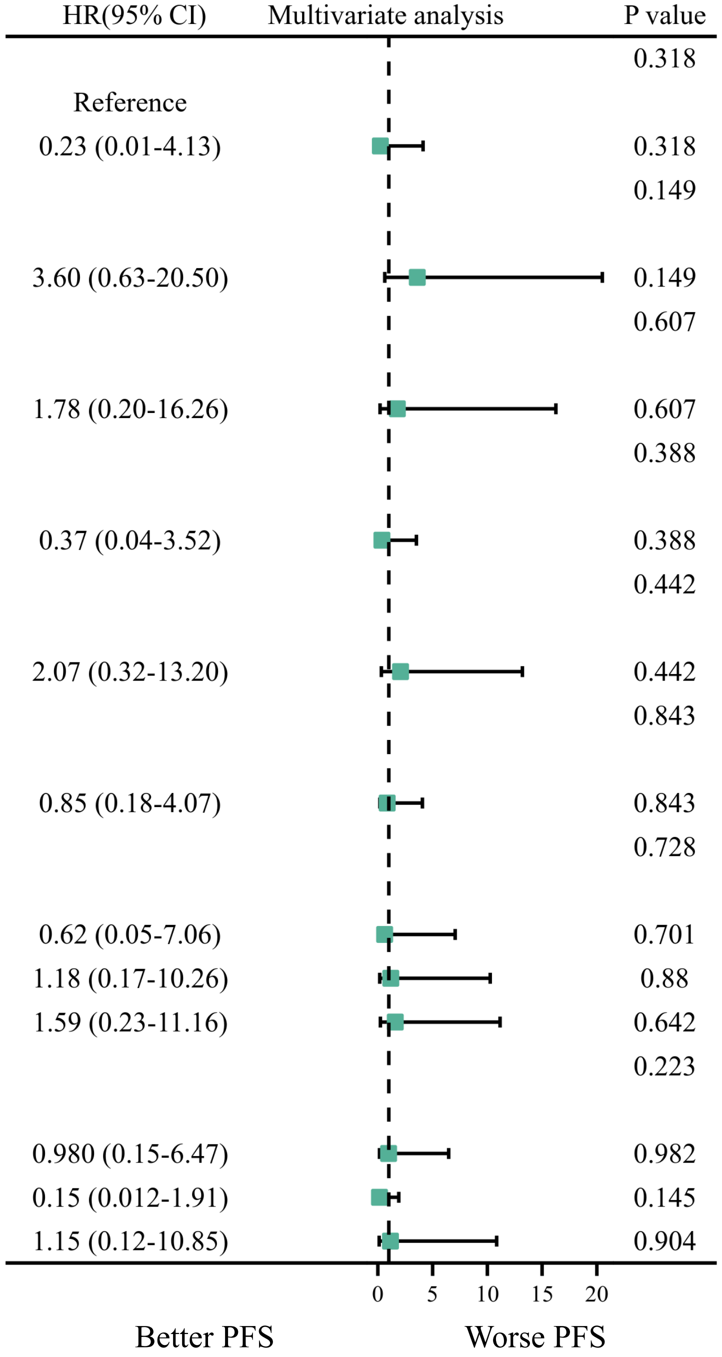

Figure S7. Case vignette illustrating the treatment outcome of patient NO.60

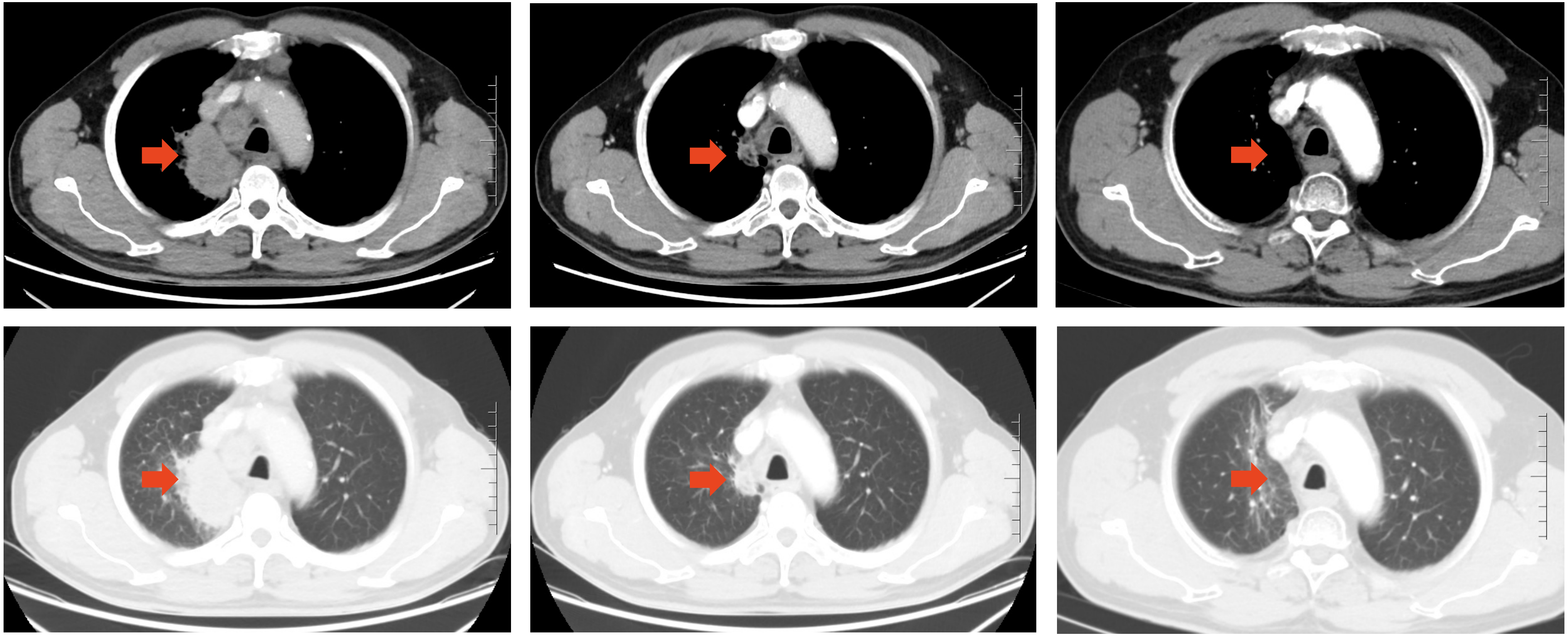

Before Treatment | After treatment 2 months | After treatment 12 months

Representative case showing the treatment response of a patient with stage IV large-cell carcinoma (Patient number 60) who received first-line pembrolizumab plus chemotherapy and achieved partial response. Computed tomography images at baseline (left panels), after receiving two cycles of the regimen middle panels), and after 12 months from initiating the regimen (right panels). Red arrows denote the primary lesion.

Figure S8. Case vignette illustrating the treatment outcome of patient NO.59.

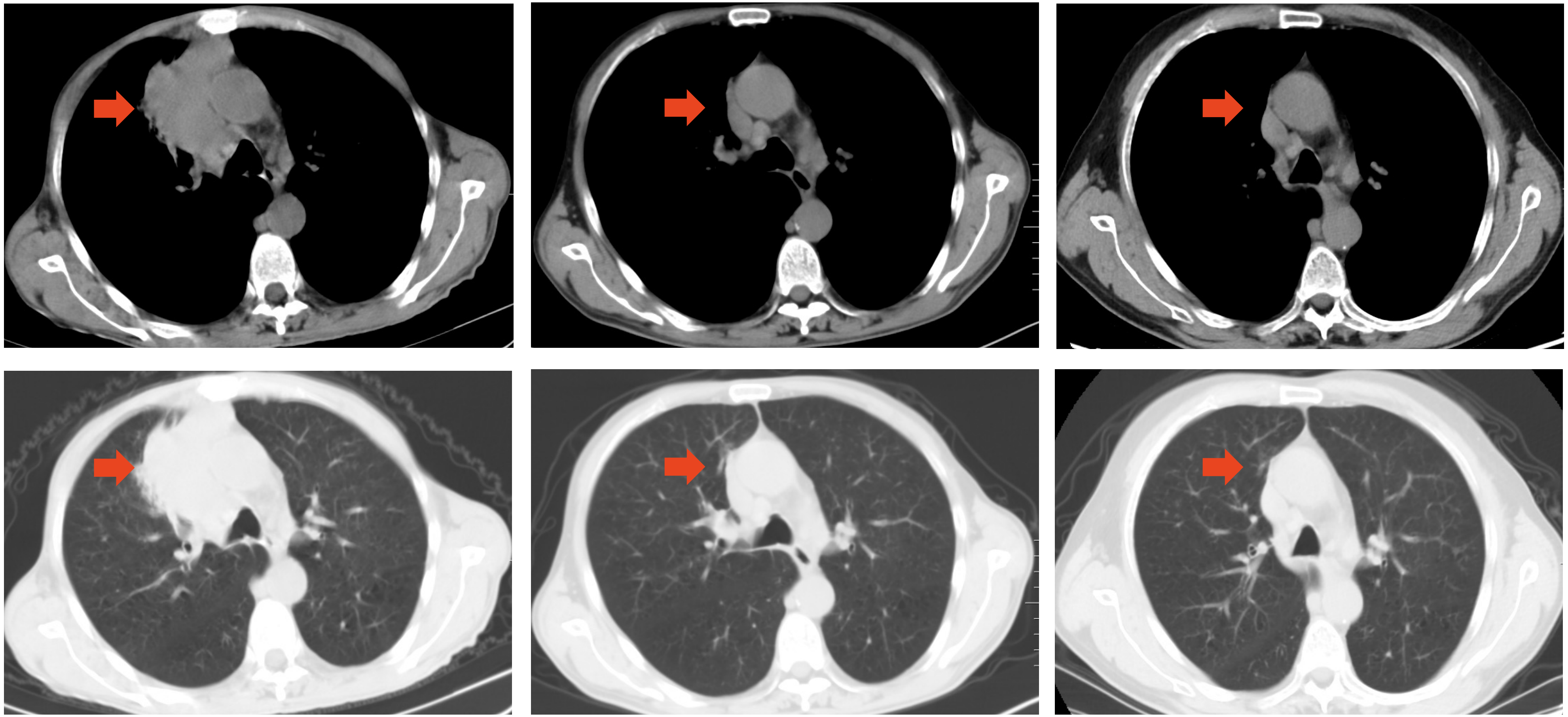

Before Treatment | After treatment 2 months | After treatment 4 months

Representative case showing the treatment response of a patient with stage IV large-cell neuroendocrine carcinoma (Patient number 59) who received first-line pembrolizumab plus chemotherapy and achieved partial response. Computed tomography images at baseline (left panels), after receiving two cycles of the regimen (middle panels), and after 4 months from initiating the regimen (right panels). Red arrows denote the primary lesion.

Table S1. Detailed information of the 60 patients with large cell lung cancer or large cell neuroendocrine carcinoma who received pembrolizumab with or without chemotherapy.

NO	Gender	Age	Smoking status	Pathology Diagnosis	Stage	p63/p40	TTF1/CD56	Syn/Napsin-A	Ki-67	PD-L1 (22C3)	Treatment line	Treatment regimen	Best Response	PFS (months)	OS (months)
P1	F	39	Never	LCC	IV	–	–	–	0.4	Unknown	2	Chemo+Pembrolizumab	PD	1.9	4.3
P2	M	58	Former	LCC	IV	–	–	–	0.8	Unknown	1	Chemo+Pembrolizumab	PD	2	5
P3	M	65	Former	LCC	IV	–	–	–	0.4	0	1	Chemo+Pembrolizumab	PD	1	1
P4	M	31	Former	LCC	IV	–	–	–	0.2	5	3	Pembrolizumab	PD	1	1
P5	M	63	Former	LCC	IV	–	–	–	0.2	35	1	Chemo+Pembrolizumab	PD	1.5	2
P6	M	76	Former	LCNEC	IV	–	–	++	0.4	Unknown	3	Pembrolizumab	PD	1.4	2.5
P7	M	65	Never	LCC	IV	–	–	–	0.7	0	2	Chemo+Pembrolizumab	PD	1	1
P8	M	58	Former	LCNEC	IV	–	–	+++	0.3	0	1	Chemo+Pembrolizumab	PD	1.5	7+
P9	M	56	Former	LCNEC	IV	–	–	+++	0.6	10	2	Chemo+Pembrolizumab	PD	2	5
P10	M	52	Former	LCC	IV	–	–	–	0.8	50	2	Chemo+Pembrolizumab	PD	1.5	1.5
P11	M	62	Never	LCC	IV	–	–	–	0.8	0	2	Chemo+Pembrolizumab	PD	1.3	9+
P12	M	55	Former	LCC	IV	–	–	–	0.7	0	2	Chemo+Pembrolizumab	PD	2	2
P13	M	55	Former	LCC	IV	–	–	–	0.4	Unknown	2	Chemo+Pembrolizumab	PD	1.4	3.5
P14	F	49	Never	LCNEC	IV	–	–	++	0.8	Unknown	2	Pembrolizumab	SD	7.6	9.5+

P15	M	63	Never	LCNEC	IV	−	−	+	0.7	0	2	Chemo+Pembrolizumab	SD	3	8
P16	M	66	Never	LCC	IV	−	−	−	0.6	90	1	Chemo+Pembrolizumab	SD	6	11+
P17	M	64	Former	LCC	IV	−	−	−	0.6	10	1	Chemo+Pembrolizumab	SD	10+	10+
P18	M	54	Former	LCC	IV	−	−	−	0.3	70	1	Chemo+Pembrolizumab	SD	3	5
P19	M	67	Former	LCC	IV	−	−	−	0.6	2	1	Chemo+Pembrolizumab	SD	2+	2+
P20	M	54	Former	LCC	IV	−	−	−	0.8	0	2	Chemo+Pembrolizumab	SD	3	3
P21	M	53	Former	LCC	IV	−	−	−	0.8	Unknown	3	Pembrolizumab	SD	6	9
P22	F	65	Never	LCC	IV	−	−	−	0.2	1	1	Chemo+Pembrolizumab	SD	14	24
P23	M	54	Never	LCNEC	IV	−	−	++	0.6	Unknown	2	Chemo+Pembrolizumab	SD	6+	6+
P24	M	71	Former	LCC	IIIb	−	−	−	0.7	30	1	Chemo+Pembrolizumab	SD	4.5	13
P25	M	64	Former	LCC	IIIb	−	−	−	0.7	Unknown	1	Chemo+Pembrolizumab	SD	1.5+	1.5+
P26	M	55	Former	LCNEC	IV	−	−	++	0.9	0	2	Chemo+Pembrolizumab	SD	8+	8+
P27	M	73	Former	LCC	IV	−	−	−	0.6	0	2	Chemo+Pembrolizumab	SD	4.5	4.5
P28	M	70	Former	LCNEC	IV	−	−	+	0.5	0	3	Chemo+Pembrolizumab	SD	3.8	3.8+
P29	M	47	Former	LCNEC	IV	−	−	+++	0.3	Unknown	1	Chemo+Pembrolizumab	SD	4	8
P30	M	57	Former	LCNEC	IV	−	−	++	0.9	6	3	Chemo+Pembrolizumab	SD	22+	22+

P31	M	60	Former	LCC	IV	−	−	−	0.6	0	1	Chemo+Pembrolizumab	SD	4	5
P32	M	66	Former	LCC	IV	−	−	−	0.6	5	1	Chemo+Pembrolizumab	SD	2+	2+
P33	M	67	Former	LCC	IV	−	−	−	0.4	90	3	Chemo+Pembrolizumab	SD	2	12.2
P34	M	60	Former	LCNEC	IV	−	−	++	0.7	Unknown	1	Chemo+Pembrolizumab	SD	4.7	11.1
P35	F	64	Never	LCC	IV	−	−	−	0.6	60	1	Chemo+Pembrolizumab	SD	2+	2+
P36	M	65	Never	LCC	IIIb	−	−	−	0.3	Unknown	1	Chemo+Pembrolizumab	SD	6	11+
P37	M	65	Former	LCC	IIIb	−	−	−	0.8	90	1	Chemo+Pembrolizumab	SD	3+	3+
P38	M	71	Former	LCC	IV	−	−	−	0.8	40	1	Chemo+Pembrolizumab	PR	2+	6+
P39	M	67	Former	LCC	IV	−	−	−	0.5	0	2	Pembrolizumab	PR	3.5	21.5
P40	M	63	Never	LCC	IV	−	−	−	0.1	0	1	Chemo+Pembrolizumab	PR	5	9+
P41	M	56	Former	LCC	IV	−	−	−	0.7	Unknown	2	Chemo+Pembrolizumab	PR	6.5	14.5
P42	M	61	Never	LCNEC	IV	−	−	+++	0.9	20	1	Chemo+Pembrolizumab	PR	7	13
P43	M	73	Former	LCC	IV	−	−	−	0.6	50	2	Pembrolizumab	PR	17	19+
P44	M	59	Former	LCC	IV	−	−	−	0.8	0	1	Chemo+Pembrolizumab	PR	10	11+
P45	M	46	Former	LCC	IV	−	−	−	0.85	0	1	Chemo+Pembrolizumab	PR	4+	4+
P46	M	66	Former	LCNEC	IV	−	−	+++	0.7	0	1	Chemo+Pembrolizumab	PR	4	13

P47	M	79	Former	LCC	IV	−	−	−	0.4	12	1	Chemo+Pembrolizumab	PR	3+	3+
P48	M	74	Former	LCNEC	IIIb	−	−	++	0.5	Unknown	1	Chemo+Pembrolizumab	PR	6+	12+
P49	M	73	Former	LCNEC	IV	−	−	++	0.5	Unknown	1	Chemo+Pembrolizumab	PR	5.5	9.5+
P50	M	64	Former	LCC	IV	−	−	−	0.8	Unknown	1	Chemo+Pembrolizumab	PR	4+	4+
P51	M	63	Former	LCNEC	IIIc	−	−	+++	0.3	Unknown	1	Chemo+Pembrolizumab	PR	12.1	33.5
P52	M	65	Former	LCC	IV	−	−	−	0.3	60	1	Chemo+Pembrolizumab	PR	15+	15+
P53	M	69	Former	LCNEC	IIIb	−	−	+++	0.4	20	1	Chemo+Pembrolizumab	PR	7.9+	7.9+
P54	M	54	Former	LCC	IV	−	−	−	0.7	0	1	Chemo+Pembrolizumab	PR	7	13+
P55	M	66	Never	LCC	IV	−	−	−	0.8	90	1	Chemo+Pembrolizumab	PR	24+	24+
P56	M	55	Former	LCC	IV	−	−	−	0.5	0	3	Chemo+Pembrolizumab	PR	14	22
P57	M	64	Former	LCC	IIIb	−	−	−	0.6	90	1	Chemo+Pembrolizumab	PR	31+	31+
P58	M	64	Former	LCC	IIIc	−	−	−	0.6	60	1	Chemo+Pembrolizumab	PR	13+	13+
P59	M	63	Former	LCNEC	IV	−	−	++	0.4	1	1	Chemo+Pembrolizumab	PR	15.3+	15.3+
P60	M	64	Former	LCC	IV	−	−	−	0.6	60	1	Chemo+Pembrolizumab	PR	35+	35+

Abbreviations: 1L, first-line; 2L, second-line; 3L, third-line; F, female; M, male; OS, overall survival; PD, progressive disease; PD-L1 TPS (22C3), programmed death- ligand 1 tumor proportion score (clone 22C3); PFS, progression-free survival; PR, partial response; SD, stable disease; WT, wild-type

Table S2. Detailed treatment history for 23 patients with large cell carcinoma (LCC) or large cell neuroendocrine carcinoma (LCNEC) who received ≥second-line pembrolizumab with or without chemotherapy.

NO	Pathology / Diagnosis	ICI treatment line	ICI treatment regimen received	Regimen received as 1L treatment	PFS (months)	Best response	Progression mode	Regimen received as 2L treatment	PFS (months)	Best response	Progression mode
P1	LCC	2L	Chemo+Pembrolizumab	TC	4	SD	Lung	–	–	–	–
P4	LCC	3L	Pembrolizumab	PC+BEV+radiotherapy	20	PR	Brain	DC+BEV	13	SD	Brain
P6	LCNEC	3L	Pembrolizumab	TC	4	SD	Adrenal gland	–	–	–	–
P7	LCC	2L	Chemo+Pembrolizumab	TC	1.7	PD	Lung	–	–	–	–
P9	LCNEC	2L	Chemo+Pembrolizumab	TC+BEV	7	PR	Lung	–	–	–	–
P10	LCC	2L	Chemo+Pembrolizumab	TC	1.2	PD	Bone, Brain, Adrenal gland	–	–	–	–
P11	LCC	2L	Chemo+Pembrolizumab	TC+BEV+radiotherapy	12	PR	Lung	–	–	–	–
P12	LCC	2L	Chemo+Pembrolizumab	PC	1	PD	Lung	–	–	–	–
P13	LCC	2L	Chemo+Pembrolizumab	TC	5	SD	Liver	–	–	–	–
P14	LCNEC	2L	Pembrolizumab	TC	5.5	SD	Lung	–	–	–	–
P15	LCNEC	2L	Chemo+Pembrolizumab	EC	4	SD	Brain	–	–	–	–
P20	LCC	2L	Chemo+Pembrolizumab	TC+radiotherapy	7	SD	Lung	–	–	–	–
P21	LCC	3L	Pembrolizumab	GC	2	PD	Lung, Bone	NC	4	SD	Lung
P23	LCNEC	2L	Chemo+Pembrolizumab	TC+BEV	5	SD	Brain, lymph nodes	–	–	–	–
P26	LCNEC	2L	Chemo+Pembrolizumab	EC	7.5	SD	Lung	–	–	–	–

P27	LCC	2L	Chemo+Pembrolizumab	TC	1	PD	Lung, lymph nodes	-	-	-	-
P28	LCNEC	3L	Chemo+Pembrolizumab	TC+BEV	2.5	SD	Lung, Brain	DC	2	PD	Brain
P30	LCNEC	3L	Chemo+Pembrolizumab	EC	2	PD	Lung, Bone	TC	2	PD	Lung
P33	LCC	3L	Chemo+Pembrolizumab	TC	4	PR	Lung, Bone	DC	4	SD	Lung
P39	LCC	2L	Pembrolizumab	TC	3	SD	Liver	-	-	-	-
P41	LCC	2L	Chemo+Pembrolizumab	NC	3	SD	Lung	-	-	-	-
P43	LCC	2L	Pembrolizumab	PC	2	PD	Lung	-	-	-	-
P56	LCC	3L	Chemo+Pembrolizumab	EC	6	SD	Brain	IC	5	SD	Lung

Abbreviations: 1L, first-line; 2L, second-line; 3L, third-line; BEV, bevacizumab; DC, docetaxel cisplatin; EC, Etoposide carboplatin; GC, gemcitabine cisplatin; IC, irinotecan carboplatin; NC, vinorelbine carboplatin; PC, Pemetrexed carboplatin; PD, progressive disease; PFS, progression-free survival; PR, partial response; SD, stable disease; TC, paclitaxel cisplatin.
